# 14-3-3 Mediates Histone Cross-Talk during Transcription Elongation in *Drosophila*

**DOI:** 10.1371/journal.pgen.1000975

**Published:** 2010-06-03

**Authors:** Caline S. Karam, Wendy A. Kellner, Naomi Takenaka, Alexa W. Clemmons, Victor G. Corces

**Affiliations:** 1 Department of Biology, Emory University, Atlanta, Georgia, United States of America; 2 Department of Biology, Johns Hopkins University, Baltimore, Maryland, United States of America; Max-Planck-Institute of Immunobiology, Germany

## Abstract

Post-translational modifications of histone proteins modulate the binding of transcription regulators to chromatin. Studies in *Drosophila* have shown that the phosphorylation of histone H3 at Ser10 (H3S10ph) by JIL-1 is required specifically during early transcription elongation. 14-3-3 proteins bind H3 only when phosphorylated, providing mechanistic insights into the role of H3S10ph in transcription. Findings presented here show that 14-3-3 functions downstream of H3S10ph during transcription elongation. 14-3-3 proteins localize to active genes in a JIL-1–dependent manner. In the absence of 14-3-3, levels of actively elongating RNA polymerase II are severely diminished. 14-3-3 proteins interact with Elongator protein 3 (Elp3), an acetyltransferase that functions during transcription elongation. JIL-1 and 14-3-3 are required for Elp3 binding to chromatin, and in the absence of either protein, levels of H3K9 acetylation are significantly reduced. These results suggest that 14-3-3 proteins mediate cross-talk between histone phosphorylation and acetylation at a critical step in transcription elongation.

## Author Summary

Activation of gene expression is thought to be regulated mainly at the level of transcription initiation. Nevertheless, many genes in *Drosophila* and vertebrates contain RNA polymerase that has started transcription but is paused 30–40 bp from the initiation site. Activation of these genes may thus be regulated by releasing the polymerase from the paused state rather than bringing this protein to the promoter. This release requires the recruitment of specialized proteins that modify the polymerase. It appears that the recruitment of these proteins takes place by modification of two chromatin proteins, histones H3 and H4. Here we characterize a process composed of multiple steps required for release of RNA polymerase from the paused state. The process starts with the recruitment of an enzyme that adds a phosphate group to histone H3. This phosphate serves as a signal to recruit a different protein, which in turn recruits a second enzyme capable of adding an acetyl group to the same histone molecule. The multiple steps involved may provide a variety of mechanisms to control the process.

## Introduction

The ability of cells to modulate their transcriptional program in response to physiological stimuli is vital for the proper development of eukaryotic organisms. This is partly achieved by post-translational modification of histone proteins that associate with the DNA to form nucleosomes, the basic units of chromatin. Histone modifying enzymes respond to signaling cues by acetylating, methylating or phosphorylating specific amino acids of histone N-termini. Such modifications alter the affinity and accessibility of chromatin to transcription activators and silencers and consequently dictate the expression profiles of genes [1]. Several transcription regulators are known to have chromatin-binding motifs that interact with histones modified at specific amino acids. One example is the chromodomain, which binds methylated histones. It was identified in the heterochromatin protein HP1 and the developmental regulator Polycomb (Pc), which bind histone H3 when methylated at lysine 9 (H3K9me) and lysine 27 (H3K27me), respectively [2]. Other regulators such as the chromatin remodeling enzyme Brd4 possess a bromodomain that recognizes acetylated histones [3]. More recently, the basal transcription factor TFIID was shown to bind H3 when methylated at K4 (H3K4me) via the plant homeodomain (PHD) finger of the subunit TAF3 [4]. Cross-talk between histone modifications adds another layer of complexity. In yeast, histone H2B monoubiquitination is required for both H3K4 and H3K79 methylation during transcription activation [5]. In *Drosophila*, a decrease in H3S10 phosphorylation leads to the ectopic spread of H3K9 methylation on polytene chromosomes [6]. Furthermore, phosphorylation of H3S10 during mitosis in mammalian cells antagonizes the effect of H3K9 methylation and leads to the dissociation of HP1 from chromosomes [7].

In addition to defining distinct transcription and chromatin states, histone modifications can also mark different stages of transcription of active genes. During the transcription process, RNA polymerase II (Pol II) communicates with histone modifying enzymes via its C-terminal domain (CTD), which consists of a heptad repeat with serine residues at positions 2 and 5. These serine residues are differentially phosphorylated depending on the specific phase of the transcription cycle. Ser5 is phosphorylated during the very early steps of elongation, while Ser2 phosphorylation marks the release of Pol II from promoter-proximal pausing, a tightly regulated checkpoint that ensures proper capping of the mRNA. In yeast, the SET 1 enzyme responsible for H3K4 methylation interacts with the C-terminal domain of Pol II when it is phosphorylated at Ser5. Consistent with this observation, H3K4 methylation is predominant at the 5′end of genes [8]. On the other hand, the SET 2 H3K36 methyltransferase associates with the Ser2-phosphorylated form of the CTD and H3K36 methylation is enriched at the 3′end of genes [9].

Phosphorylation of histone H3 at Ser10 (H3S10ph) has long been implicated in transcription activation in organisms ranging from yeast to humans [10]–[12] but it was only more recently that a mechanistic explanation for how H3S10ph could contribute to gene activation became apparent. Mahadevan and colleagues showed that several members of the 14-3-3 phospho-binding protein family interact with H3 only when phosphorylated and that human 14-3-3ζ is recruited to *c-fos* and *c-jun* when these genes are transcriptionally activated [13]. Furthermore, Winter et al. showed that 14-3-3 is recruited to the HDAC1 gene upon its activation in an H3S10ph-dependent manner and is required for the transcription of this gene [14]. More recently, Zippo et al. have shown that 14-3-3 plays a crucial role in the transcription of the mammalian FOSL1 gene by recruiting the histone acetyltransferase MOF. Acetylation of H4K16 by MOF helps recruit the double bromodomain BRD4 protein, which in turn recruits P-TEFb [15]. These authors suggest that H3K9ac is also involved in the process but evidence and details of the proteins involved are not available.

The *Drosophila* genome contains two different 14-3-3 genes, 14-3-3ζ and 14-3-3ε, providing a simple model to study 14-3-3 function. The role and dynamics of H3S10ph during transcription activation in *Drosophila* are well characterized. Phosphorylation of H3 during interphase is carried out by the JIL-1 kinase and mutations in the *JIL-1* gene lead to severe disruption of polytene chromosome morphology with a marked loss of the well-defined pattern of bands and interbands characteristic of wild-type chromosomes [16]. These structural perturbations do not affect the recruitment of transcription factors or Pol II to active genes. Instead, H3S10 phosphorylation is required during promoter-proximal pausing for recruitment of the P-TEFb kinase and phosphorylation of the CTD of Pol II at Ser2, which is required for the release of Pol II and transcription elongation [17].

Here we explore the role of H3S10ph- mediated recruitment of 14-3-3 to chromatin during transcription activation. Results show that 14-3-3 proteins are recruited to active genes in a JIL-1-dependent manner and are required for phosphorylation of Pol II at Ser2. Further analyses indicate that 14-3-3 interacts with elongator protein Elp3, a histone acetyltransferase required during early elongation. The recruitment of Elp3 to chromatin and the subsequent acetylation of H3K9 are dependent on JIL-1, suggesting that 14-3-3 proteins mediate crosstalk between H3 phosphorylation and acetylation during early transcription elongation.

## Results

### JIL-1–mediated H3 phosphorylation is required during early transcription elongation

Numerous studies in organisms ranging from yeast to vertebrates have established a role for H3S10 phosphorylation in transcription activation [10]–[12]. Specifically, studies in *Drosophila* indicate that this modification is involved in the release of Pol II from promoter-proximal pausing during early transcription elongation [17]. The JIL-1 kinase, which is the homologue of the vertebrate MSK1/2 kinases, is responsible for this modification in *Drosophila* [16]. Interestingly, mutations in *JIL-1* not only result in a genome-wide decrease in transcription but also cause dramatic changes in the structure of polytene chromosomes [16]. Although the two effects are probably related, it has been recently questioned whether JIL-1 and H3S10 phosphorylation play a role in transcription and whether the observed recruitment of JIL-1 to heat-shock genes upon induction, and the ensuing H3S10 phosphorylation, are artifacts resulting from the fixation procedures utilized in the immunofluorescence microscopy analyses used to derive these conclusions [18]. To address these concerns, JIL-1 antibodies were used in standard chromatin immunoprecipitation (ChIP) experiments to examine whether JIL-1 is recruited to the *hsp70* promoter when the gene is induced in *Drosophila* Kc cells. The results confirm our previous observations showing that JIL-1 binds to the promoter region of the *hsp70* gene only after the cells are subjected to heat-shock (Supporting Figure 1A in Text S1). The same result was observed in ecdysone-induced genes after hormone stimulation (data not shown). Total levels of H3K79 methylation and H3K36 methylation, which mark transcription elongation, were also examined and found to be reduced in *JIL-1* mutants, providing additional and independent evidence for our previous finding of a role for JIL-1 in transcription elongation (Supporting Figure 1B and 1C in Text S1). Recruitment of JIL-1 to the *hsp70* promoter explains the phosphorylation of H3S10 at heat-shock puffs, which has been observed consistently by various investigators using antibodies from different sources and varying fixation protocols [11], [17], [19]–[23]; also see Supporting Figure 4A in Text S1. In agreement with this conclusion, polytene chromosomes from *JIL-1* null mutants lack H3S10ph at the heat-shock puffs, while Pol II phosphorylated at Ser5 is still present [17]. Taken together, these findings strongly support a role for JIL-1 in transcription elongation in general and in that of *hsp70* in particular. Results presented below as well as those of Zippo et al., 2009 lend further support to this conclusion.

### 14-3-3 proteins localize to actively transcribed regions on polytene chromosomes

**Figure 1:**
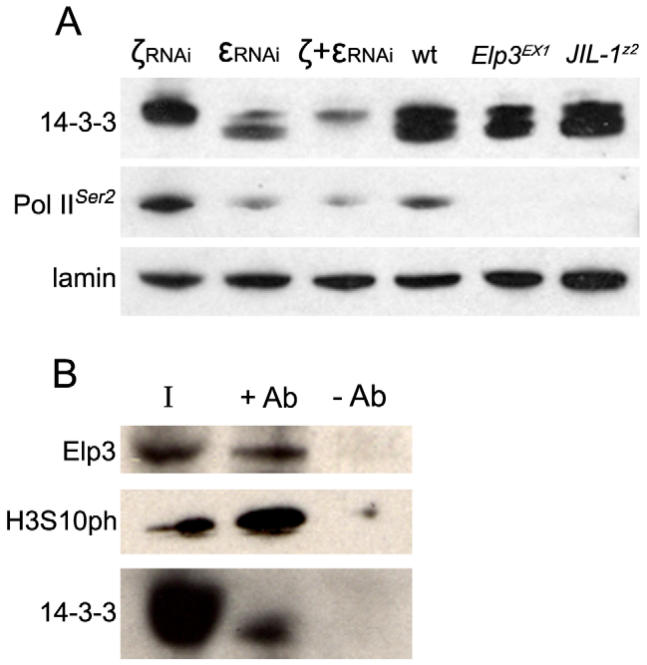
14-3-3 proteins are required for transcription elongation of most *Drosophila* genes and interact with the elongation protein Elp3. (A) Western analysis of 14-3-3 proteins, Pol IIo^ser2^ and lamin in salivary glands from wild type (wt) larvae, *JIL-1* ^Z2^, *Elp3^EX1^* mutant larvae, or salivary glands expressing RNAi against 14-3-3ζ (ζRNAi), 14-3-3ε (εRNAi), or both simultaneously (ζ+εRNAi). (B) Western analysis of Kc cell extract immuno-precipitated with 14-3-3 antibodies (+Ab) or beads alone (-Ab) and probed for Elp3, 14-3-3 and H3 phosphorylated at S10 (H3S10ph). I =  input.

In order to better understand the mechanisms underlying transcription regulation by JIL-1 mediated H3S10 phosphorylation and to elucidate downstream events, the role of 14-3-3 proteins in transcription was examined. Previous studies have shown that two human 14-3-3 proteins (14-3-3ζ and 14-3-3ε) associate with active genes in an H3S10ph-dependent manner [13]–[15]. However, these studies were limited to the analysis of specific genes and it is yet unclear whether 14-3-3 plays a more general role in transcription. A pan 14-3-3 antibody was used to examine whether 14-3-3 proteins are broadly distributed on *Drosophila* polytene chromosomes. This antibody was raised against the peptide DKSELVQKAKLAEQAERY found in the N-terminus of human 14-3-3β, which is highly similar to an amino acid stretch in *Drosophila* 14-3-3ζ (DKEELVQKAKLAEQSERY) and partially so to *Drosophila* 14-3-3ε (ERENNVYKAKLAEQAERY) [24]. When protein extracts from salivary glands of third instar wild type larvae are analyzed on Western blots using this antibody, two bands of approximately 25 kDa can be seen (Figure 1A). A hs-Gal4 strain that expresses Gal4 in the salivary glands in the absence of any heat-shock treatment [25] was then used to express UAS-RNAi against 14-3-3ζ, 14-3-3ε, or both simultaneously in order to determine the identity of the two bands (Figure 1A). When RNAi against 14-3-3ζ is expressed, the lower band is severely diminished, with significant increase in the signal from the higher band. When RNAi against 14-3-3ε is expressed, a severe reduction of the higher band is observed, accompanied by partial reduction of the lower band. RNAi against both isotypes leads to a reduction in both bands, suggesting that the antibody recognizes both isotypes, and that the higher band corresponds to 14-3-3ε while the lower one corresponds to 14-3-3ζ (Figure 1A).

**Figure 2:**
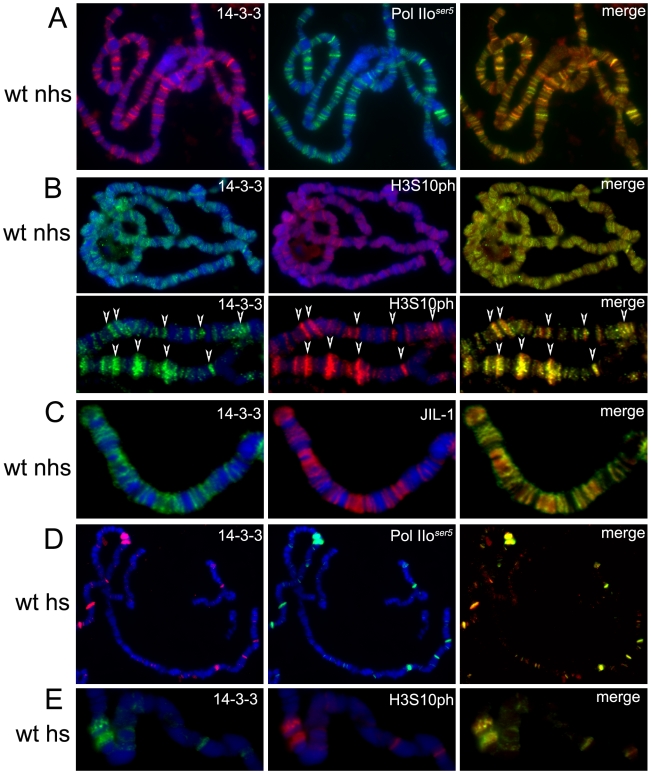
14-3-3 proteins localize to actively transcribed regions on polytene chromosomes and colocalize with H3S10P and JIL-1. Immuno-colocalization of 14-3-3 with Pol IIo^ser5^ (A,D), H3S10ph (B,E) or JIL-1 (C) on polytene chromosomes of wild type (wt) larvae. (nhs =  not heat-shocked, hs =  after heat-shock), DAPI (blue) stains DNA. (B) Includes close up of representative chromosome region for better depiction of colocalization (arrows).

This antibody was then used in immunofluorescence microscopy to determine the localization of 14-3-3 proteins on polytene chromosomes derived from salivary glands of *Drosophila* third instar larvae. The results reveal a broad distribution of 14-3-3 proteins on chromatin as depicted in Supporting Figure 2A in Text S1. This signal is dramatically decreased in larvae expressing RNAi against both isoforms of 14-3-3, verifying the specificity of the antibody in immunofluoresence analyses (Supporting Figure 2B in Text S1); antibodies against the insulator-binding protein Su(Hw) were used to control for signal intensity. Co-staining with anti-Pol IIo^ser5^ (Figure 2A) shows that 14-3-3 proteins are found at interband regions and significantly co-localize with Pol II, as reflected by the abundance of yellow signal in the merge panel (see close up in Figure 2A). Similar analyses using anti-H3S10ph (Figure 2B) and anti-JIL-1 (Figure 2C) antibodies show that 14-3-3 also co-localizes extensively with these two proteins, consistent with the hypothesis that they bind phosphorylated histones.

The heat-shock paradigm was then used to better understand the dynamics of 14-3-3 distribution during transcription. When larvae are subjected to temperature elevation, the heat-shock (*hsp*) genes are turned on while all genes that were previously active are turned off. Immunofluorescence analyses of polytene chromosomes using antibodies against H3S10ph show that heat-shock treatment leads to disappearance of the modification from previously active genes and its redistribution to the induced *hsp* genes [11]. If 14-3-3 is recruited by phosphorylation of H3S10, the distribution of 14-3-3 should exhibit similar behavior to that of H3S10ph upon heat-shock. Third instar larvae were incubated at 36.5°C for 20 min and their salivary glands were immediately dissected and fixed for immunofluorescence analysis using anti-14-3-3 in combination with anti-Pol IIo^ser5^ (Figure 2D) or anti-H3S10ph (Figure 2E) antibodies. Results show that the pattern of 14-3-3 binding, like that of H3S10 phosphorylation, changes from a broad distribution throughout the genome to one that is restricted to the heat shock genes. This suggests that 14-3-3 proteins are recruited to actively transcribed genes and that their binding to chromatin correlates with H3S10 phosphorylation.

### 14-3-3 binding to chromosomes is dependent on H3S10 phosphorylation and is required for transcription elongation

**Figure 3:**
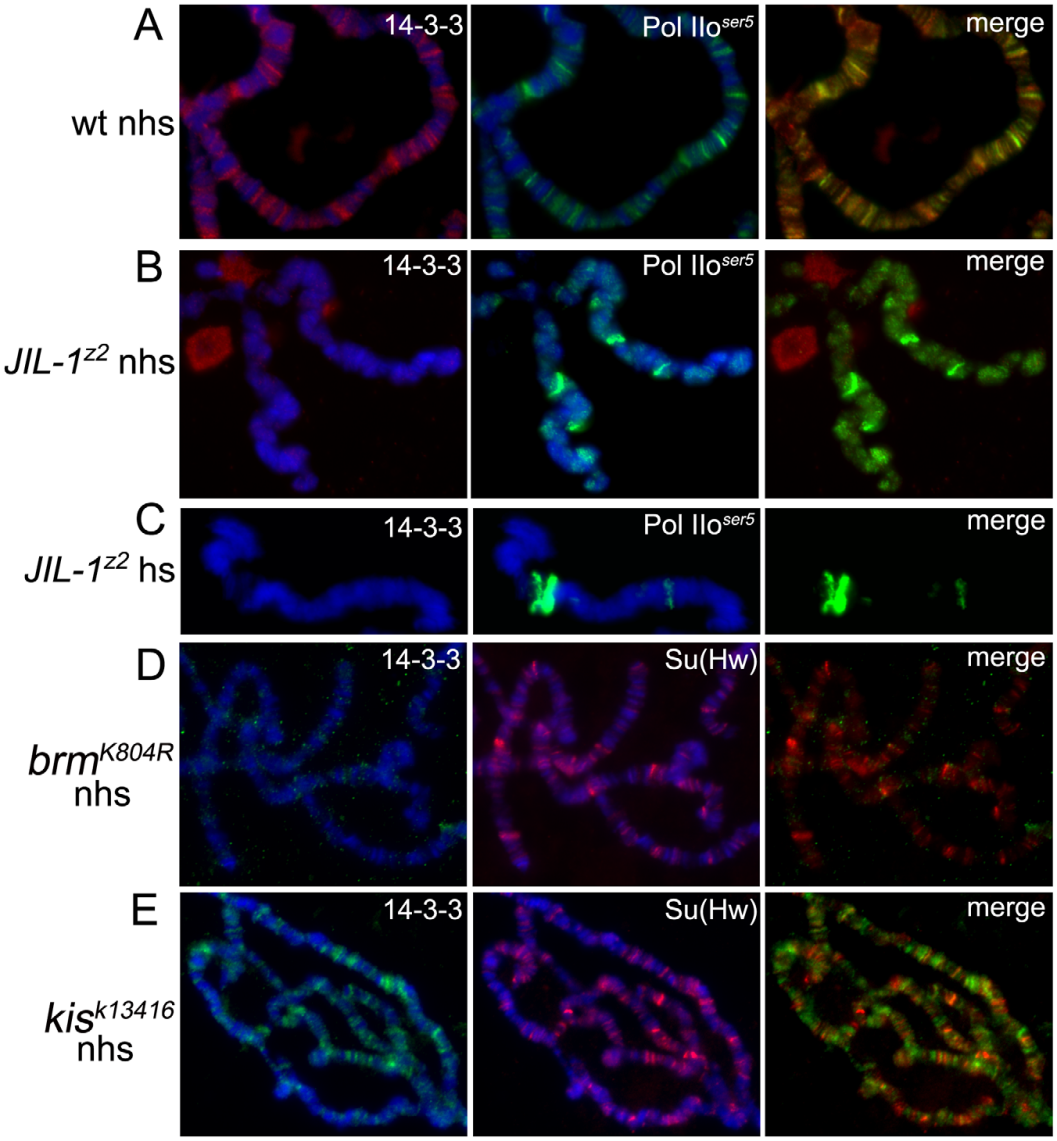
Binding of 14-3-3 to polytene chromosomes is dependent on phosphorylation of H3S10 by JIL-1. Immunolocalization of 14-3-3 and Pol IIo^ser5^ on wild type (A), *JIL-1* mutant (B,C) polytene chromosomes before (A,B) and after (C) heat-shock. Immunolocalization of 14-3-3 and Su(Hw) on polytene chromosomes from *brm* mutants (D) or *kis* mutants (E). (nhs =  not heat-shocked, hs =  after heat-shock). DAPI (blue) stains DNA.

The experiments described above were then repeated in *JIL-1^z2^* null mutants to determine whether the recruitment of 14-3-3 is dependent on H3S10 phosphorylation. We have previously reported that despite the disrupted structure of the chromosomes of these mutants, Pol IIo^ser5^ can still be detected on chromatin at wild-type levels [17]. Antibodies against Pol IIo^ser5^, as well as Su(Hw), were therefore used to control for signal intensity levels. As can be seen in Figure 3B and 3C, and Supporting Figure 2C in Text S1, no 14-3-3 protein can be detected on the chromosomes of *JIL-1^z2^* mutants before or after heat-shock as compared to wild type (Figure 3A, and Supporting Figure 2C in Text S1) whereas levels of Pol IIo^ser5^ (Figure 3B–3C) and Su(Hw) (Supporting Figure 2C in Text S1) are unaffected. This is not due to a decrease in total levels of 14-3-3 in the cell, as they remain unchanged in *JIL-1^z2^* mutants (Figure 1A). The binding of 14-3-3 to chromosomes was also examined in salivary glands that express a dominant negative form of the Brahma (BRM) chromatin remodeling ATPase, which acts very early during transcription initiation of many genes – excluding heat-shock genes – and is required for the recruitment of the Pol II machinery to chromosomes [25]; levels of phosphorylated H3S10 are significantly reduced in chromosomes from larvae carrying this dominant mutation [17]. Results indicate that the binding of 14-3-3 to polytene chromosomes is also disrupted in the dominant negative *brm* mutant, supporting the conclusion that H3S10 phosphorylation is required for the presence of 14-3-3 on chromatin (Figure 3D). In contrast, similar analyses on chromosomes from heat-shocked *brm* mutants show that 14-3-3 is recruited to heat-shock puffs (Supporting Figure 2D in Text S1), consistent with the previous report that BRM is not required for activation of heat-shock genes [26].

Taken together, the findings described above establish a correlation between H3S10 phosphorylation, 14-3-3 recruitment and transcription. It remains possible, however, that 14-3-3 proteins bind to other components of the transcription machinery and that their association with H3S10 phosphorylation is strictly correlative and indirect. 14-3-3 recruitment to polytene chromosomes was therefore examined in larvae that are mutant for the *kismet* (*kis*) gene, which encodes a chromatin remodeling ATPase required during early elongation of transcription. In these mutants, like in *JIL-1* mutants, transcription can initiate but productive elongation is impeded [27]. H3S10ph levels, however, remain comparable to wild-type [17]. As can be seen in Figure 3E, the binding of 14-3-3 to chromatin in *kis* mutants is similar to that observed in wild-type larvae, consistent with the hypothesis that 14-3-3 binding is specifically dependent on H3S10 phosphorylation and not simply on transcription elongation.

Given the broad distribution of 14-3-3 proteins on polytene chromosomes as well as their close association with H3S10 phosphorylation, we hypothesized that 14-3-3 proteins may play a general role in transcription elongation. Western analysis was therefore used to determine levels of elongating Pol II in salivary gland cells expressing RNAi against each of the *Drosophila* 14-3-3 proteins separately or both simultaneously. As can be seen in Figure 1A, RNAi against 14-3-3ε alone or both 14-3-3 proteins leads to a significant decrease of total Pol II^ser2^ levels, while RNAi against 14-3-3ζ has no effect. Similarly, levels of H3K36 methylation, a marker of transcription elongation, were significantly reduced when both proteins were knocked down (Supporting Figure 1C in Text S1). Anti-lamin antibodies were used to control for loading in both experiments.

### The Elongator protein Elp3 associates with 14-3-3 *in vivo* and is dependent on JIL-1 for binding to chromosomes

14-3-3 proteins function as versatile dimeric structures that can modulate various forms of protein-protein interaction in response to signaling cues. In many instances they serve as scaffolds, bridging proteins that cannot directly interact [28]. We therefore hypothesized that the recruitment of 14-3-3 to chromatin by phosphorylated H3S10 may serve to regulate the interaction between H3S10ph and other chromatin-binding proteins. Such proteins would have to (1) interact with 14-3-3, (2) be chromatin-related, and (3) function during transcription elongation. Various reported biochemical screens, aimed at isolating 14-3-3-binding proteins, provide a vast database to search for candidates that fit these criteria. An exhaustive review of 14-3-3 interactors identified the histone acetyltransferase Elp3 (Elongator protein 3) [29], [30], a subunit of the Elongator complex that co-purifies with Pol II [31] and is required for H3 acetylation specifically during transcription elongation in yeast [29], [32], [33].

**Figure 4:**
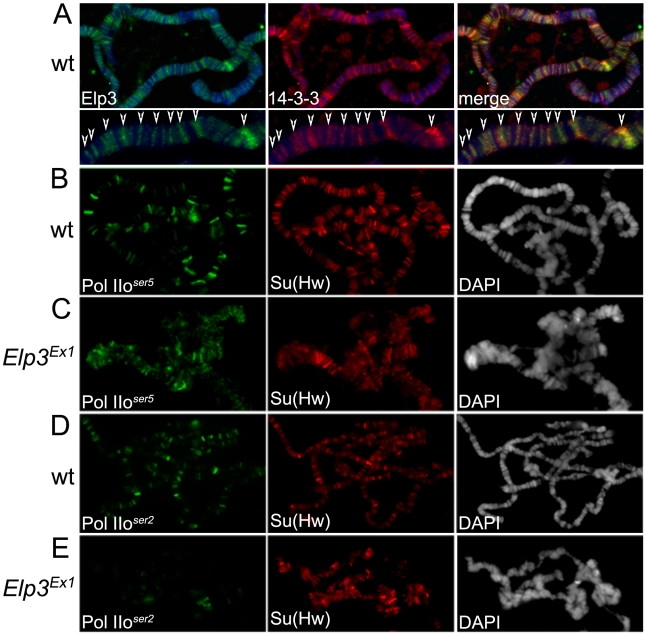
Elp3 co-localizes with 14-3-3 and is required for proper chromatin structure and transcription elongation. (A) Immunolocalization of Elp3 and 14-3-3 on wild type (wt) polytene chromosomes. Close up of representative chromosome region for better depiction of colocalization (arrows). DAPI (blue) stains DNA. (B–E) Immunolocalization of Pol IIo^ser5^ (B,C) or Pol IIo^ser2^ (D,E) on polytene chromosomes of wild type (wt) (B,D) or *Elp3* mutant (C,E) larvae. Su(Hw) was used as internal control for signal intensity. DAPI is shown in grey to depict chromatin structure.

In order to confirm an interaction between Elp3 and 14-3-3 in *Drosophila*, anti-14-3-3 antibodies were used to immunoprecipitate proteins from Kc cell extracts followed by Western analysis using anti-Elp3 antibodies. Figure 1B shows that both Elp3 and phosphorylated H3S10 co-precipitate when 14-3-3 anti-serum is used but not with beads alone, suggesting that these proteins directly or indirectly interact *in vivo*. The distribution of Elp3 on chromosomes and its co-localization with 14-3-3 was also examined. As expected from a histone acetyltransferase that is involved in active transcription, Elp3 is found at interband regions when analyzed by immunofluorescence microscopy (Figure 4A, left panel). Co-staining with 14-3-3 revealed co-localization between the proteins at many sites (see representative close up), suggesting that the interaction observed by co-immunoprecipitation may be relevant to their role in transcription.

**Figure 5:**
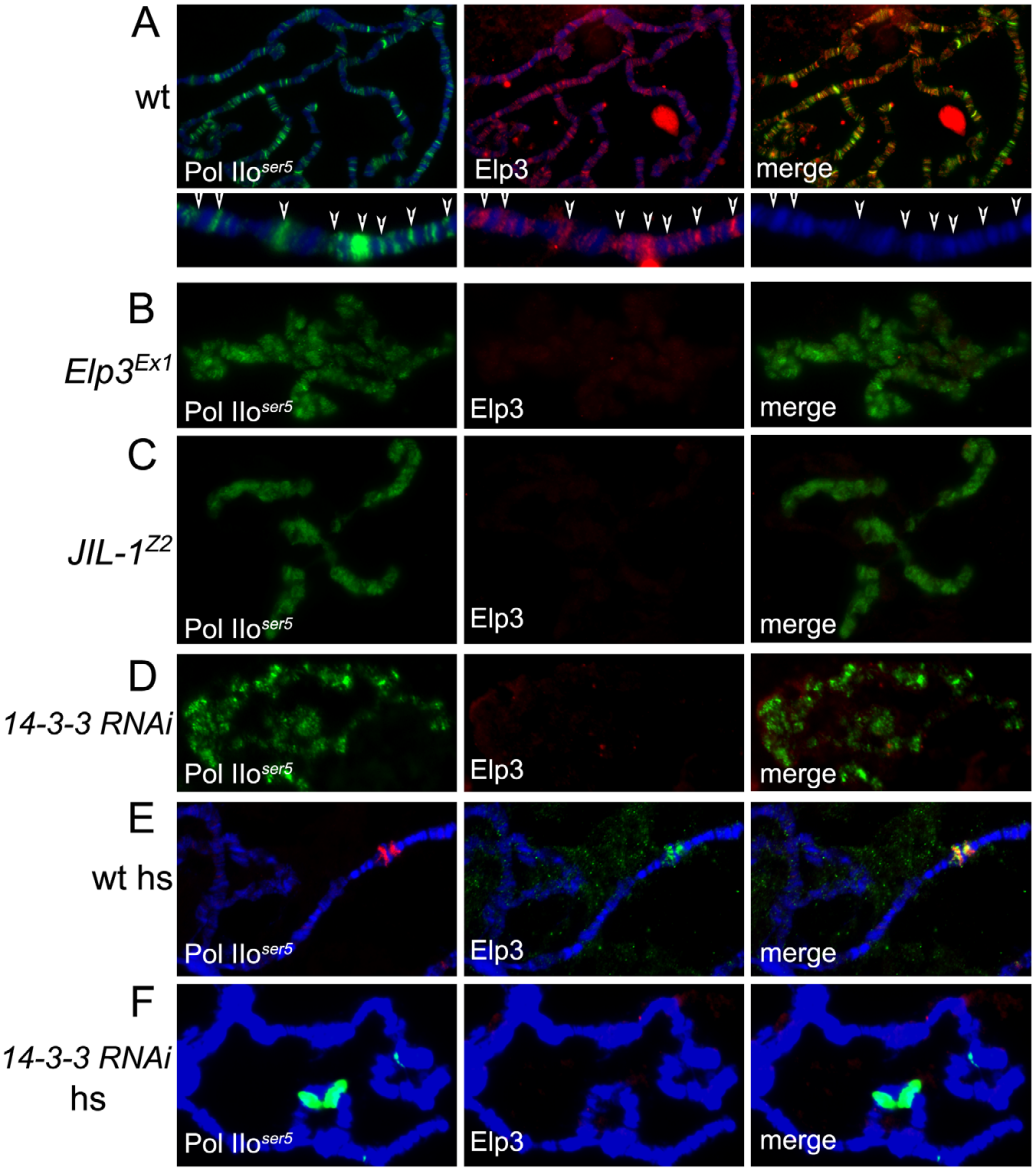
JIL-1 and 14-3-3 are required for Elp3 binding to chromosomes. (A–F) Immunolocalization of Elp3 and Pol IIo^ser5^ on polytene chromosomes from wild type (wt)(A,E), *Elp3* mutant (B) or *JIL-1^Z2^* mutant (C) larvae or larvae expressing 14-3-3 RNAi (D,F). (A) includes close up of representative chromosome region for better depiction of localization of Elp3 to interbands and colocalization with Pol II (arrows). (hs =  after heat-shock).

We then asked whether the role of Elp3 in transcription elongation is conserved in *Drosophila*. The Elp3 protein is present at heat-shock puffs after heat-shock (Figure 5E), suggesting it is recruited to active genes upon induction. The *Elp3^EX1^* allele was generated by imprecise excision of a P-element inserted 65 bp upstream of the transcription start site creating a deletion from the P-insertion site to the triplet encoding K277 [34]. This mutation results in pupal lethality and formation of melanotic tumors during larval stages. Since the mutant flies are unable to make any Elp3 protein, the late lethality is probably a consequence of the perdurance of maternal Elp3 protein into pupal stages of development. Therefore, larvae carrying the *Elp3^EX1^* allele may still accumulate some Elp3 protein and they cannot be considered null [34]. This is especially obvious in the morphology of the polytene chromosomes, which present a range of chromatin alterations from complete absence of interbands to slightly distorted chromosomes still showing some band/interband pattern (data not shown). We thus selected chromosomes with fully penetrant phenotypes such as those shown in Figure 4 and Figure 5 because they lack Elp3 protein (see Figure 5B) and are representative of a null effect. Chromosomes with some remnant of the band/interband pattern still contain some Elp3 protein and were not considered in our analysis. Levels of Pol II binding to chromatin were analyzed by immunofluorescence imaging of polytene chromosomes using antibodies to Su(Hw) as an internal control. Both anti-Pol IIo^ser5^ and anti-Pol IIo^ser2^ antibodies were used to determine whether flies carrying *Elp3* mutations exhibit transcription defects. Consistent with a role in elongation, levels of Pol IIo^ser5^ are maintained at wild type levels in the *Elp3^EX1^* mutant (compare Figure 4B and 4C) while those of Pol IIo^ser2^ were dramatically reduced (compare Figure 4D and 4E). Western analysis of protein extracts from salivary glands of *Elp3^EX1^* mutants also show that total levels of Pol IIo^ser2^ are severely reduced when compared to wild type (Figure 1A). Levels of methylated H3K36, a marker of transcription elongation, are also reduced in these mutants, consistent with a role for Elp3 in transcription (Supporting Figure 1C in Text S1).

If 14-3-3 recruits Elp3 in response to H3S10 phosphorylation, we would expect to see less Elp3 protein on polytene chromosomes in the absence of 14-3-3 or *JIL-1*. To determine if this is the case, antibodies against Elp3 and phosphorylated Pol II ^ser5^ were used to stain polytene chromosomes from wild type and *JIL-1* mutant larvae or larvae expressing RNAi against both 14-3-3 mRNAs. Elp3 co-localizes with Pol II^ser5^ at interband regions in wild type chromosomes (Figure 5A, representative close up). In contrast, no Elp3 protein can be detected on chromosomes of *JIL-1* mutants or 14-3-3 RNAi knock-down when Pol II^ser5^ (Figure 5C and 5D) or Su(Hw) (Supporting Figure 3C–3F in Text S1) were used as internal controls. This is most evident at heat-shock puffs, where levels of Elp3 proteins are significantly reduced when 14-3-3 protein levels are knocked down (Figure 5F) as compared to wild type (Figure 5E). At the same time, JIL-1 protein can still bind to chromosomes in *Elp3* mutants (Supporting Figure 3B in Text S1) at wild type levels (Supporting Figure 3A in Text S1), suggesting that Elp3 functions downstream of JIL-1 during transcription elongation. In agreement with this conclusion, JIL-1 and H3S10ph are present at heat shock puffs after temperature elevation in polytene chromosomes from *Elp3* mutant larvae (data not shown).

### JIL-1–dependent recruitment of Elp3 is required for acetylation of H3K9

**Figure 6:**
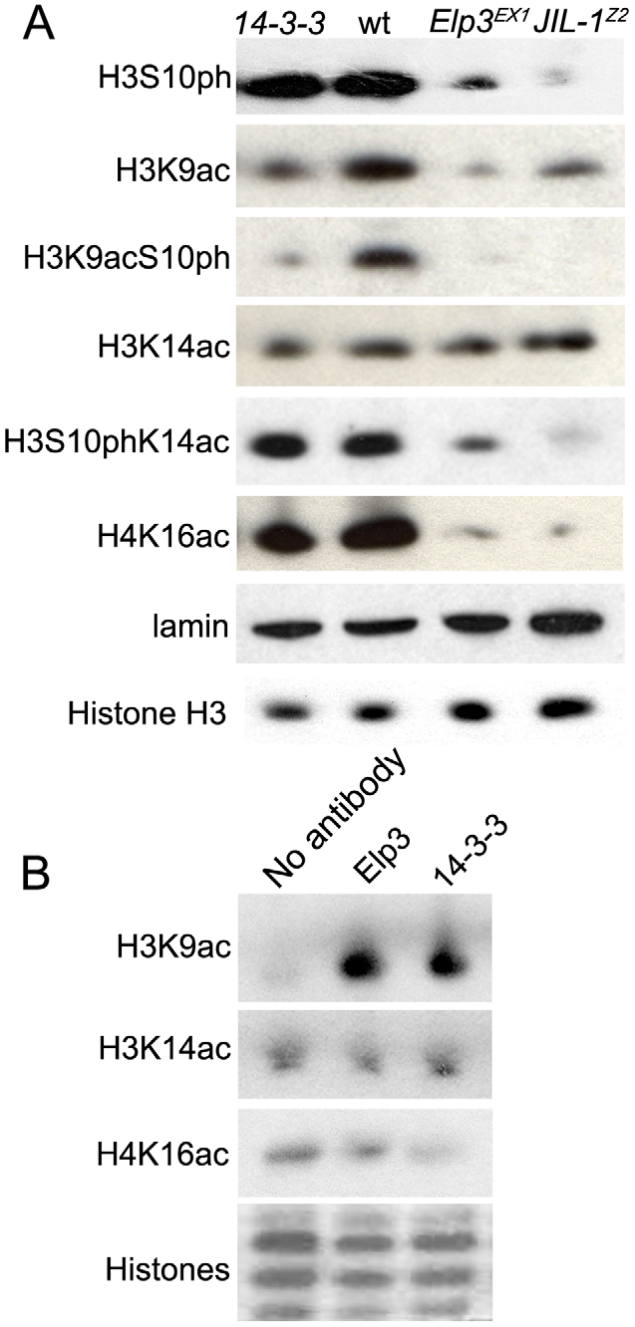
JIL-1, 14-3-3, and Elp3 are required for H3 acetylation. (A) Western analysis of levels of: phosphorylated H3S10 (H3S10ph), acetylated H3K9 (H3K9ac), phosphoacetylated H3K9S10 (H3K9acS10ph), acetylated H3K14 (H3K14ac), phosphoacetylated H3S10K14 (H3S10phK14ac), acetylated H4K16Ac (H4K16ac), total H3 and lamin in salivary gland cell extracts of wild type (wt), *Elp3^EX1^*, or *JIL-1^Z2^* mutant larvae or those expressing RNAi against both 14-3-3 isoforms (14-3-3). (B) Western analysis of acetylation levels of recombinant histones (H3K9ac, H3K14ac, or H4K16ac) subjected to an *in vitro* histone acetylase assay using larval lysates that were co-immunoprecipitated with either anti-Elp3 or anti-14-3-3 antibodies. Coomassie stain shown as loading control.

The above results point to a functional interaction between JIL-1 (an H3 kinase) and Elp3 (an H3 acetyltransferase), suggesting crosstalk between histone H3 phosphorylation and acetylation during transcription elongation. H3 has been shown to be acetylated at K14 at induced heat-shock genes [11], [21]. Immunofluorescence analysis of polytene chromosomes from heat-shocked wild type larvae detected a strong signal at heat-shock puffs when H3S10phK14ac antibodies were used (Supporting Figure 4A in Text S1), suggesting that the two modifications occur on the same histone tails. A similar analysis also detected H3K9 acetylation at the heat-shock genes (Supporting Figure 4B in Text S1). While we were unable to detect any signal using H3K9acS10ph antibodies by immunofluorescence analysis of polytene chromosomes, Western blot analysis shows that H3K9ac and H3S10ph also occur on the same histone tail *in vivo* (Figure 6A). We first used these different antibodies to confirm that Elp3 acts as a histone acetyltransferase in *Drosophila*. Antibodies against H3K9ac and H3K14ac were used in Western blot analyses to compare H3 acetylation levels in *Elp3* mutant and wild type larvae. As can be seen in Figure 6A, levels of H3K9ac are significantly diminished in *Elp3* mutants, while those of H3K14ac are unaffected. In addition, immunofluorescence analysis with anti-H3K9ac shows that levels of this modification are diminished in polytene chromosomes of *Elp3* mutants (Supporting Figure 5D in Text S1). To further confirm the specificity of *Drosophila* Elp3 as a histone H3K9 acetyltransferase, we isolated Elp3 by immunoprecipitation and used the protein in an *in vitro* acetylation assay using recombinant histones as a substrate. The results of these experiments show significant acetylation of H3K9 as compared to the no antibody control (Figure 6B). Consistent with the data from Western analysis, acetylation of H3K14 could not be detected above background in this *in vitro* assay (Figure 6B).

Interestingly, levels of H3S10ph are slightly reduced in *Elp3* mutants (Figure 6B), despite the fact that JIL-1 binding to chromosomes is not affected (Supporting Figure 3B in Text S1). Antibodies to phospho-acetylated H3K9S10 and phospho-acetylated H3S10K14 were then used to determine the extent of the doubly modified histone H3 tails in *Elp3* mutants. As can be seen in Figure 6A, mutations in *Elp3* affect H3K9acS10ph to a larger extent than H3S10phK14ac as expected from the differential effects observed on the acetylation of the two Lys residues.

Since JIL-1 is required for the recruitment of Elp3, it follows that the H3K9 acetylation defect observed in *Elp3* mutants would also be observed in the absence of JIL-1 and 14-3-3 proteins. Western analyses of protein extracts derived from salivary glands from *JIL-1^z2^* mutant larvae or glands expressing RNAi against both 14-3-3 proteins (Figure 6A) and immunofluoresence analysis (Supporting Figure 5B and 5C in Text S1) show that levels of acetylated H3K9 are markedly reduced in both cases. In addition, immunoprecipitation of 14-3-3 followed by the *in vitro* acetylation assay showed that 14-3-3 associates with a histone acetyltransferase activity that is specific to H3K9 and not H3K14, similar to the effect observed with Elp3 (Figure 6B).

The results discussed above suggest that 14-3-3 proteins recruit the histone H3 acetyltransferase Elp3, which in turn acetylates H3 in the Lys9 residue. This observation is interesting in the context of recent findings suggesting that 14-3-3 can recruit the histone acetyltransferase MOF to acetylate H4K16 in the mammalian FOSL1 gene [15]. To test whether this is also the case in *Drosophila*, we determined whether levels of H4K16 acetylation are affected by downregulation of 14-3-3 using RNAi against the two genes encoding this protein in *Drosophila*. Results show that the presence of H4K16ac in protein extracts from larval tissues (Figure 6) or 3^rd^ instar polytene chromosomes (Supporting Figure 6 in Text S1) is not affected in flies lacking 14-3-3 proteins. To further confirm the absence of association between 14-3-3 and *Drosophila* MOF, we isolated 14-3-3 and associated proteins by immunoprecipitation with 14-3-3 antibodies and used the proteins in an *in vitro* acetylation assay using recombinant histones as a substrate. The results of these experiments show absence of H4K16 acetylation when compared to the no antibody control (Figure 6B). These results suggest that recruitment of MOF may not require 14-3-3 or that the function of these proteins may be redundant.

## Discussion

Covalent modification of histone tails can influence the binding of transcription regulators and accordingly modulate gene expression. The phosphorylation of histone H3 at S10 was recently shown to be accompanied by the recruitment of members of the 14-3-3 protein family to specific genes upon induction [13]–[15]. Here we extend these observations and present evidence that 14-3-3 mediates a novel histone crosstalk that promotes transcription elongation in *Drosophila*.

*Drosophila* 14-3-3 proteins display a broad distribution on polytene chromosomes, colocalizing with phosphorylated Pol II at many sites. This localization is dependent on the presence of H3S10P and, like H3S10 phosphorylation, redistributes to heat-shock genes upon temperature elevation. In addition, total levels of elongating Pol II are severely diminished in cells lacking both 14-3-3 isotypes. These findings establish a role for 14-3-3 proteins during transcription elongation of many *Drosophila* genes. The specific roles of the individual 14-3-3 isotypes, however, remain unclear. The two proteins have been shown to exhibit significant functional specificity with some aspects of redundancy across various cell processes [24], [35]–[38]. It was recently shown that while 14-3-3ε is essential for hatching of *Drosophila* embryos, *14-3-3*ε null mutants survive because one isotype of the *14-3-3ζ* gene is upregulated at the time of hatching, compensating for the loss of 14-3-3ε [39]. This, together with the data presented here, suggests that the two isotypes may play redundant roles in transcription. Total levels of active Pol II remain unaffected when 14-3-3ζ is knocked down. This could imply that 14-3-3ε is the sole isotype required for transcription elongation. However, given that *14-3-3*ε null mutants are viable [39], it is more likely that the two proteins play redundant functions in transcription, and that the upregulation of 14-3-3ε in response to 14-3-3ζ knockdown is a compensatory mechanism. Further analyses are required to determine the exact function of each isotype. Antibodies with better specificity, for example, can be used to determine whether the two proteins colocalize at all sites on chromosomes or whether they exhibit unique binding patterns. Since these proteins can function as heterodimers, it is reasonable to speculate that while they may play a basic redundant function in transcription, specific combinations of 14-3-3 binding and interaction allow to fine tune the transcription process, thereby adding an extra layer of complexity to the histone code.

From a mechanistic perspective, the data presented here suggest that 14-3-3 proteins mediate cross-talk between histone phosphorylation and acetylation during transcription elongation by creating a bridge between JIL-1 and Elp3. In yeast, Elp3 is a subunit of the Elongator complex, which was first identified by its association with elongating Pol II [31], and later shown to be involved in histone acetylation and transcription [29]; results here show that these functions are conserved in *Drosophila*. On the other hand, most Elp3 protein is cytoplasmic, and a few studies have implicated the complex in tRNA modification [40], [41]. It is therefore possible that the mutant effects documented here are indirect and can be alternatively explained by translation defects. However, our results point to a direct role for Elp3 in transcription. Elp3 binds to transcriptionally active regions on polytene chromosomes and is recruited to heat-shock genes after heat-shock. It interacts with 14-3-3 and co-localizes with it at many sites on polytene chromosomes. More importantly, binding of Elp3 to chromosomes is dependent on JIL-1 and 14-3-3. Consistent with these observations, decreased levels of H3K9 acetylation are detected in *JIL-1* mutants and 14-3-3 knockdowns, suggesting that acetylation by Elp3 is dependent on phosphorylation by JIL-1 and the subsequent recruitment of 14-3-3. Taken together, these results strongly support a direct role for histone acetylation by Elp3 during transcription elongation, downstream of JIL-1 and 14-3-3.

Interestingly, it was recently shown in mammals that 14-3-3 proteins bind phosphorylated H3S10 at the FOSL1 gene and serve to recruit the H4K16 acetyltransferase MOF, which then acetylates histone H4 at the Lys16 residue and is required for recruitment of BRD4 and P-TEFb. Although the specific aspects of the process have not been explored in detail, it appears that recruitment of BRD4 in mammals also requires acetylation of H3K9 [15]. Consistent with this observation, we have previously shown that phosphorylation of H3S10 is required for recruitment of P-TEFb to heat-shock genes in *Drosophila* [17]. Nevertheless, it appears that in *Drosophila* 14-3-3 does not play a major role in the recruitment of MOF; instead, 14-3-3 recruits Elp3 and this protein is then required for MOF recruitment, based on the observation that H4K16Ac is dramatically reduced in flies carrying a mutation in the *Elp3* gene. Taken together, the data strongly support a role for crosstalk between histone phosphorylation and acetylation during the release of Pol II from promoter-proximal pausing.

The relationship between histone H3 phosphorylation and acetylation has been the subject of some debate. These two modifications are known to occur in response to the same stimuli, in the same tissue and on the same histone tails. Two possible models have been consequently put forward to explain these observations [42]. The first proposes that the two modifications are synergistic and coupled such that one is dependent on the other. This is supported by the fact that, *in vitro*, HATs preferentially acetylate histone tails that are phosphorylated, suggesting that histone phosphorylation provides a stronger binding site for HATs than unphosphorylated ones [43]. The second model envisions the two modifications being performed by regulatory machineries that are recruited simultaneously yet independently to active genes [42]. Studies that support this model have utilized specific antibodies that recognize the modifications either individually or together. Two populations of histones were detected at active genes using these antibodies, a larger highly acetylated population that is not phosphorylated and a smaller phosphoacetylated population, suggesting that phosphorylation is not a prerequisite to acetylation [44], [45]. Our results support the ‘synergistic and coupled’ model, but under the premise that 14-3-3 acts as a bridge, rather than one modification acting as a binding site for the next enzyme. At the same time, the data do not rule out the other scenario. In fact, while different groups have in the past advocated one model over the other, the two are by no means mutually exclusive. It is becoming increasingly evident that multiple layers of regulation come into play during transcription activation. Promoter-proximal pausing, rather than initiation, appears to be the rate limiting step in the activation of a significant number of genes in humans and *Drosophila* [46]–[48]. Along the same lines and more pertinent to this study, there are at least two reports of distinct pathways responsible for histone acetylation during gene activation in yeast: Gcn5 is known to acetylate histones during transcription initiation, while Elp3 acetylates histones during elongation [32], [33]. It is therefore possible that the acetylation that occurs during initiation is not dependent on phosphorylation, while the acetylation associated with elongation is. This would account for the two different pools.

Additional evidence of a functional link between Elp3 and JIL-1 was obtained from imaging polytene chromosomes of *Elp3* mutants, which display defects in chromosome structure that are very similar to those caused by mutations in *JIL-1*. These defects are characterized by the loss of organization of band and interband regions and a shortening of the chromosome arms. What these defects signify in terms of the roles of JIL-1 and Elp3 in transcription, however, remains unclear. Further analyses will be required to determine the exact cause of the chromosome morphological defects and precisely how they affect transcription. This will help explain the molecular mechanisms governing transcription regulation by histone phosphorylation and acetylation and further shed light into the complex relationship between chromosome structure and transcription.

## Materials and Methods

### *Drosophila* stocks

Stocks were maintained in standard medium at 18°C or 25°C. Oregon R larvae were used for wild type (wt) controls in all experiments. The *JIL-1^z2^* stock was a gift from Dr. K. Johansen (Iowa State University). The *brm^K804R^* and *kis^k13416^* stocks were a gift from Dr. J. Tamkun (UC Santa Cruz). The *Elp3^EX1^* mutant was a gift from Dr. J. Svejstrup (Cancer Research UK London Research Institute). UAS-14-3-3ζ RNAi flies were obtained from VDRC (Stock #48724) and UAS-14-3-3ε RNAi flies were obtained from NIG (Stock #31196R-4). To express siRNA in salivary glands these stocks were crossed to +/+; hsp70-Gal4/hsp70-Gal4 (Bloomington, 1799). To express RNAi against both 14-3-3 isotypes simultaneously, 48724/48724; 31196R-4/31196R-4 flies were crossed to +/+; hsp70-Gal4/hsp70-Gal4.

### Preparation of *Drosophila* protein extracts and western analysis

Approximately 100 pairs of salivary glands from third instar wild type or mutant larvae or glands subject to RNAi were homogenized in 100 µl RIPA buffer with EDTA free protease inhibitors (Roche) and phosphatase inhibitors (Sigma #P2850) and left on ice for 15 min. Laemmli's buffer and beta-mercaptoethanol were added and lysates were incubated at 65°C for 20 min to solubilize proteins and then insoluble fractions were spun down. Samples were run on NuPage 4–12% gradient Bis-Tris gels and transferred to PVDF membranes for immunodetection. Membranes were incubated overnight at 4°C in antibody dilution buffer (PBS/0.05% Tween/5% milk or BSA in case of Pol II antibodies) containing primary antibodies at concentrations of 1∶1000 rabbit α-14-3-3 (SCBT), 1∶1000 mouse α-Pol IIo^ser2^ (H5, Covance), 1∶5000 mouse α-lamin C (Developmental Studies Hybridoma Bank), 1∶1000 rabbit α-H3S10P (Millipore), 1∶5000 rabbit α-H3K9Ac (Millipore, 07-352), 1∶5000 rabbit α-H3K14Ac (Millipore, 07-353), 1∶1000 rabbit α-H3S10PK9Ac (Abcam, ab12181), 1∶1000 rabbit α-H3S10PK14Ac (Millipore, 07-081), 1∶1000 rabbit α-H3K79me (Abcam) and 1∶10000 rabbit α-histone H3 (Abcam).The membranes were washed twice with PBS/0.25% Tween, incubated for 1h at room temperature in the appropriate HRP secondary antibody (Jackson ImmunoResearch Laboratories) and washed twice with PBS/0.25% Tween. Antibody signal was visualized using chemi-luminescence detection methods (SuperSignal West Pico kit, Pierce).

### Induction of the heat-shock response and analysis of polytene chromosomes

Salivary gland polytene chromosome squashes were prepared from wandering third instar larvae maintained at 18°C. For heat-shock experiments, third-instar wild type and *JIL-1^z2^* mutant larvae were subjected to heat-shock treatment as described previously (Nowak et al., 2003). Salivary glands were dissected in 0.7% NaCl and fixed for 2 min in 45% acetic acid/1.85% formaldehyde. Fixed salivary glands were subsequently squashed in 45% acetic acid on subbed slides. The slides were frozen in liquid nitrogen and stored dry at −70°C. For immunostaining of 14-3-3 proteins, salivary glands were fixed for 1 min in 3.7% acetic acid, 2 min in 45% acetic acid/3.7% formaldehyde and 3 min in 45% acetic acid. Slides were incubated overnight at 4°C in antibody dilution buffer (PBS/0.1% Triton X-100/1% BSA) containing primary antibodies at concentrations of 1∶50 α-14-3-3 (K19, SCBT), 1∶100 rabbit α-JIL-1, 1∶20 rabbit α-Elp3 [29], 1∶30 mouse α-Pol IIo^ser2^ (H5, Covance), 1∶30 mouse α-Pol IIo^ser5^ (H14, Covance), 1∶150 rat α-Su(Hw), 1∶50 rabbit α-H3S10phK14ac and 1∶50 rabbit α-H3K9ac. Following incubation, slides were washed three times in PBS/0.1% Triton X-100 and incubated for 1 h at 37°C in the appropriate secondary antibody (Jackson ImmunoResearch Laboratories) diluted 1∶200 in antibody dilution buffer. Slides were washed three times as described above and stained with 0.5 µg/ml of 4′,6-diamidino-2-phenylindole (DAPI) and mounted in Vectashield mounting medium (Vector Laboratories) for viewing.

### Co-immunoprecipitation experiments

Goat anti 14-3-3ζ serum (SCBT) was covalently crosslinked to Protein G sepharose beads 4 Fast Flow (GE Healthcare) using DMP. *Drosophila* Kc cells grown to 80% density were spun down, washed twice in PBS and crosslinked for 10 min in 1% paraformaldehyde. Cells were washed twice in PBS to stop the reaction and then lysed in RIPA buffer containing phosphatase inhibitors and protease inhibitors for 20 min on ice. The insoluble fraction was spun down and the soluble fraction split in two, half on the beads with antibody and the other half with beads but no antibody as a control; a small fraction was reserved for the input lane. The samples were run on 4–12% NuPage Bis/Tris gel, transferred to nitrocellulose, blocked in 1% BSA and blotted against rabbit α-14-3-3ζ (SCBT) 1∶2000, rabbit α-Elp3 1∶2000 [29], and mouse α-H3S10P (Millipore).

### *In vitro* acetylation assay

Wild type third instar larvae were repeatedly washed in PBS and then ground and vortexed in RIPA buffer with protease inhibitors. The lysate was diluted 10-fold with 1% Triton X-100/150 mM NaCl/50 mM Tris and spun down to eliminate insoluble fractions. The soluble lysate was incubated with either no antibody, Elp3 polyclonal antibody, or 14-3-3 polyclonal antibody and pulled down with protein G beads. The beads were washed 5 times in 1% Triton X-100 buffer and 3 times in 50 mM Tris pH8/150 mM NaCl containing protease inhibitors. To 25 µl of the beads containing pull-down, 50 µl 50 mM Tris pH8.0/150 mM NaCl, 20 µl of 1 mg/ml histones and 5 µl of 5.69 mM AcetylCoA were added per 75 µl reaction and incubated at 30°C for 45 min with mixing. The histones were then collected for Western analysis.

### Chromatin immunoprecipitation experiments

*Drosophila* Kc cells were grown at 25°C to 7×10^6^ cells/ml in serum-free HyQ-CCM3 medium (HyClone Laboratories, Inc.). Cells were subjected to heat shock by addition of an equivalent volume of medium preheated to 48°C to the growing cells. After holding the cells at 36.5°C for 15 min, the cells were immediately cooled down to 25°C with the addition of 1/3 total volume of 4°C medium immediately prior to cross-linking [49]. Cells were cross-linked with 1% formaldehyde for 10 min, quenched with 0.125 mM glycine and washed with PBS. Nuclear lysates were sonicated to generate 200–1000 bp DNA fragments. Immunoprecipitation was performed with 7 µl α-JIL-1 or with no antibody. Immunoprecipitated DNA was extracted and amplified with primers described in Boehm et al., 2003:

*hsp70*+4F, 5'-CAATTCAAACAAGCAAAGTGAACAC

*hsp70*+112R, 5'-TGATTCACTTTAACTTGCACTTTA.

## Supporting Information

Text S1 Supporting information and figures.
(2.66 MB DOC)
