# Supplementary material for: 14-3-3 Mediates Histone Cross-Talk during Transcription Elongation in Drosophila
Source: PLoS Genet. 2010 Jun 3;6(6):e1000975. doi: 10.1371/journal.pgen.1000975 (PMC2880557; doi:10.1371/journal.pgen.1000975)
Supplement: Text S1 — Supporting information and figures. (2.66 MB DOC) [file pgen.1000975.s001.doc]

**Supporting Figure 1**

(A) JIL-1 is recruited to the promoter of the *hsp70* gene after heat-shock: Chromatin immunoprecipitation of Kc cell extracts by anti-JIL1 antibodies before and after heat-shock followed by PCR using primers that amplify the promoter of the hsp70 gene.

(B) Western analysis of levels of methylated H3K79 (H3K79me) in salivary glands of wild type (wt) or *JIL-1^Z2^* mutant larvae.

(C) Western analysis of levels of methylated H3K36 (H3K36me) in salivary glands of wild type, *Elp3^EX1^*, or *JIL-1^Z2^* mutant larvae or salivary glands expressing RNAi against both 14-3-3 isoforms.

**Supporting Figure 2**

(A-D) Immunolocalization of 14-3-3 on polytene chromosomes from wild type larvae (A), larvae expressing 14-3-3 RNAi (B), *JIL-1^Z2^* mutant larvae (C) or heat-shocked *brm* mutant larvae (D). Su(Hw) (A-C) or Pol IIo^ser5^ (D) were used to control for signal intensity.

**Supporting Figure 3**

(A-B) Immunolocalization of JIL-1 on polytene chromosomes from wild type (A) or *Elp3* mutant larvae (B).

(C-F) Immunolocalization of Elp3 on polytene chromosomes from wild type (C), *Elp3* mutant larvae (D), larvae expressing 14-3-3 RNAi (E) or *JIL-1^Z2^* mutant larvae (F). Su(Hw) was used as internal control.

**Supporting Figure 4**

(A) Immunolocalization of phosphoacetylated H3S10K14 and Pol IIo^ser5^ on wild type polytene chromosomes after heat-shock.

(B) Immunolocalization of acetylated H3K9 and Pol IIo^ser5^ on wild type polytene chromosomes after heat-shock.

**Supporting Figure 5**

Immunolocalization of H3K9ac in wild type larvae (A), *JIL-1^Z2^* mutant larvae (B), larvae expressing 14-3-3 RNAi (C) or *Elp3^EX1^* mutant larvae (D). Pol IIo^ser5^ was used as an internal control.

**Supporting Figure 6**

Immunolocalization of H4K16ac in female wild type larvae (A), *JIL-1^Z2^* mutant larvae (B), larvae expressing 14-3-3 RNAi (C) or *Elp3^EX1^* mutant larvae (D). Pol IIo^ser5^ was used as an internal control.

**Supporting Figure 7**

Immunolocalization of H4K16ac in male wild type larvae (A), *JIL-1^Z2^* mutant larvae (B), larvae expressing 14-3-3 RNAi (C) or *Elp3^EX1^* mutant larvae (D). Pol IIo^ser5^ was used as an internal control.

**Supporting Figure 8**

(A) Quantification of immunofluorescence signal from anti-14-3-3 antibodies on polytene chromosomes from wild type larvae, or *JIL-1*, *kis* or *brm* mutant larvae under non heat-shock (Figures 3 A-B and D-E) or heat-shock (Figure 2D and 3C) conditions. Signal was normalized to levels of Pol IIo^ser5^ (2D, 3A-C) or Su(Hw) (D-E).

(B) Quantification of immunofluorescence signal from anti-Pol IIo^ser5^ or anti-Pol IIo^ser2^ antibodies on polytene chromosomes from wild type larvae or *Elp3* mutant larvae (Figures 4 B-E). Signal was normalized to levels of Su(Hw).

(C) Quantification of immunofluorescence signal from anti-Elp3 antibodies on polytene chromosomes from wild type larvae, larvae expressing 14-3-3 RNAi, or *Elp3*or *JIL-1* mutant larvae under non heat-shock (Figures 5 A-D) or heat-shock (Figures 5 E-F) conditions. Signal was normalized to levels of Pol IIo^ser5^.

**Supporting Figure 9**

(A) Quantification of immunofluorescence signal from anti-14-3-3 antibody staining of polytene chromosomes from wild type larvae, larvae expressing 14-3-3 RNAi or *JIL-1* mutant larvae normalized to Su(Hw) (Supporting Figures 2 A-C in Text S1).

(B) Quantification of immunofluorescence signal from anti-Elp3 antibody staining of polytene chromosomes from wild type larvae, larvae expressing 14-3-3 RNAi, or *JIL-1*or *Elp3* mutant larvae, normalized to Su(Hw) levels (Supporting Figures 3 C-F in Text S1).

(C) Quantification of immunofluorescence signal from anti-H3K9ac antibody staining of polytene chromosomes from wild type larvae, larvae expressing 14-3-3 RNAi, or *JIL-1*or *Elp3* mutant larvae, normalized to Pol IIo^ser5^ levels (Supporting Figures 5 A-D in Text S1).

(D) Quantification of immunofluorescence signal from anti-H4K16ac antibody staining of polytene chromosomes from female (left panel) or male (right panel) wild type larvae, larvae expressing 14-3-3 RNAi, or *JIL-1*or *Elp3* mutant larvae, normalized to Pol IIo^ser5^ levels (Supporting Figures 6A-D and 7A-D in Text S1).


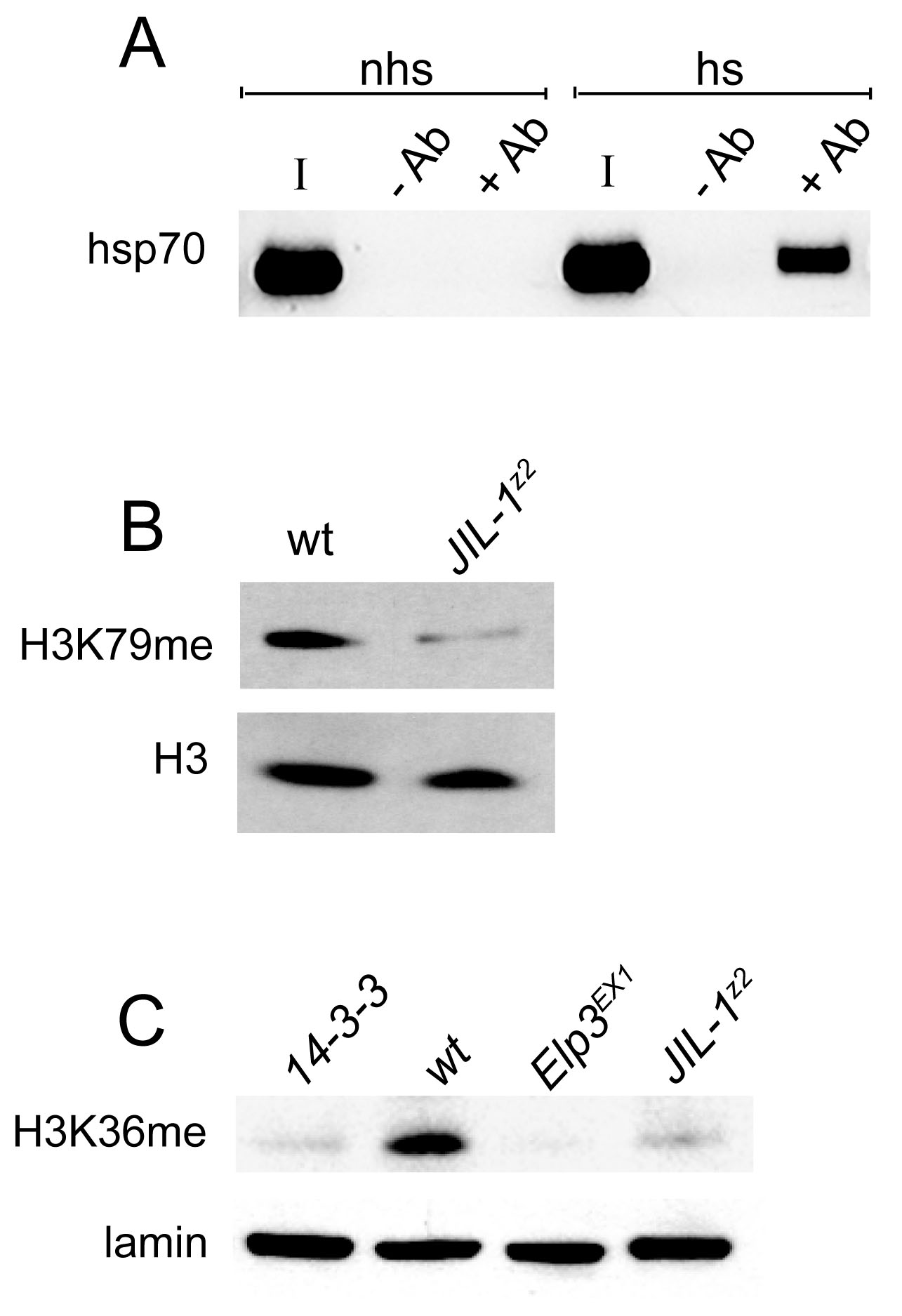


Supporting Figure 1


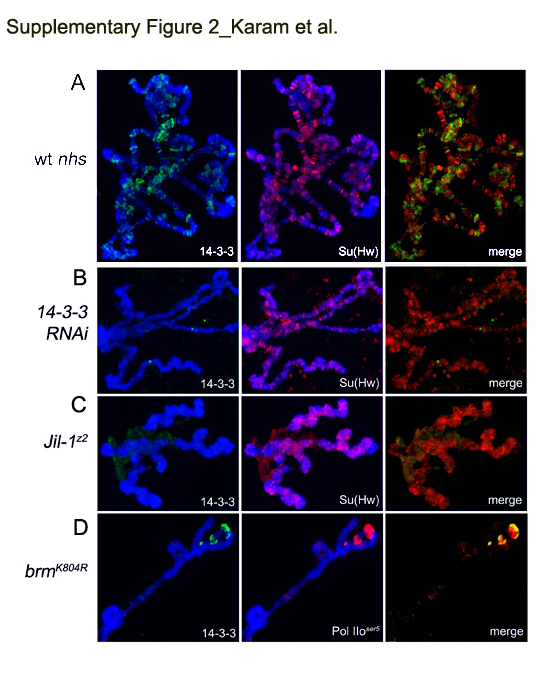


Supporting Figure 2


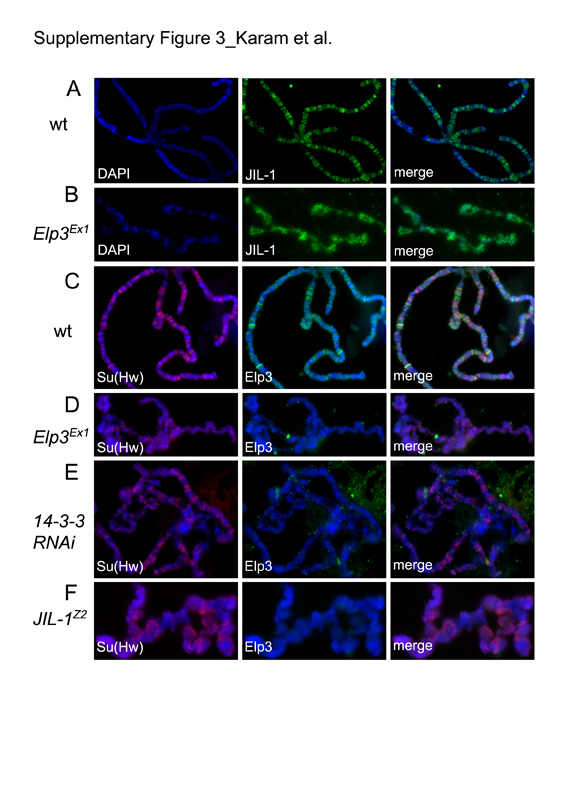


Supporting Figure 3


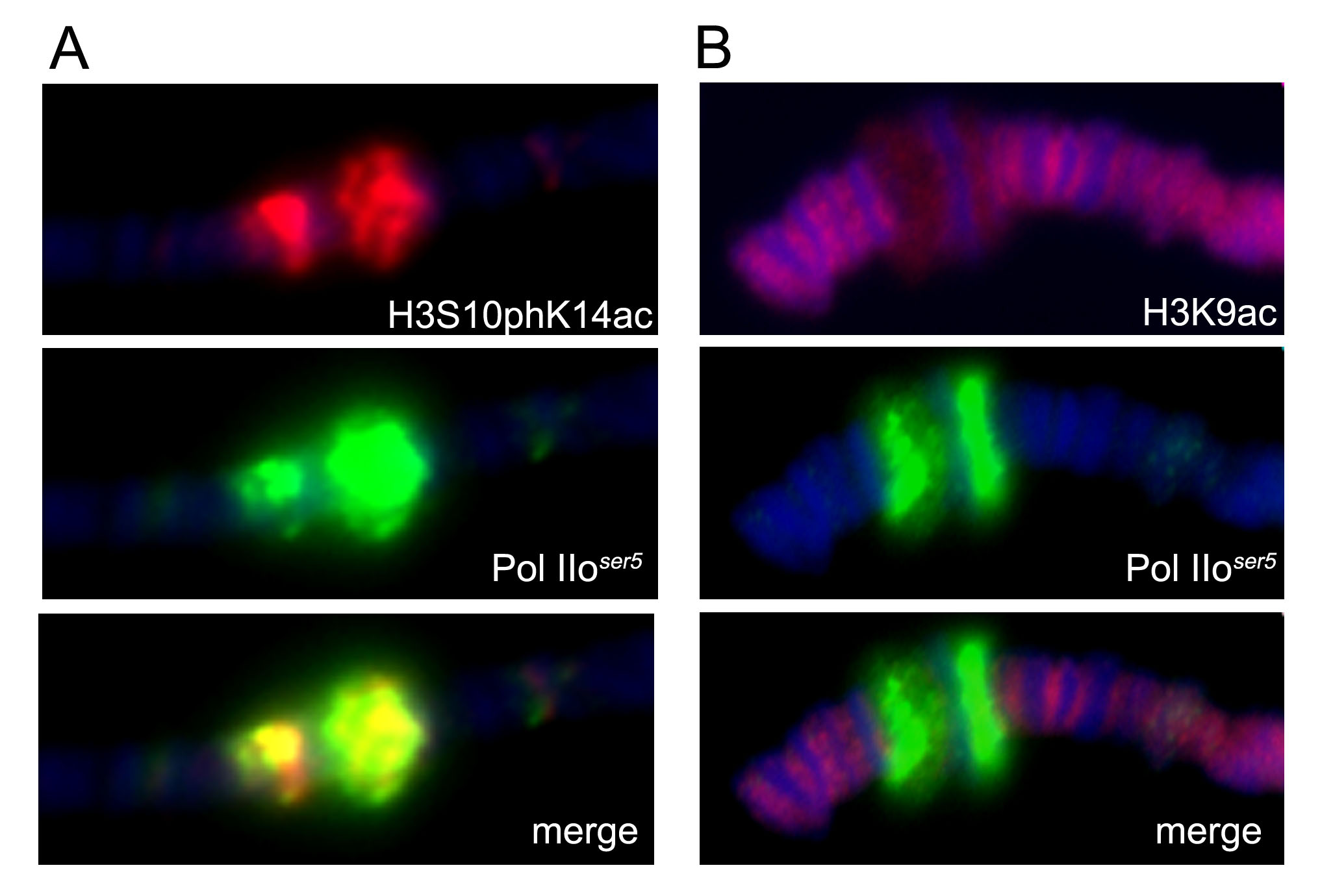


Supporting Figure 4


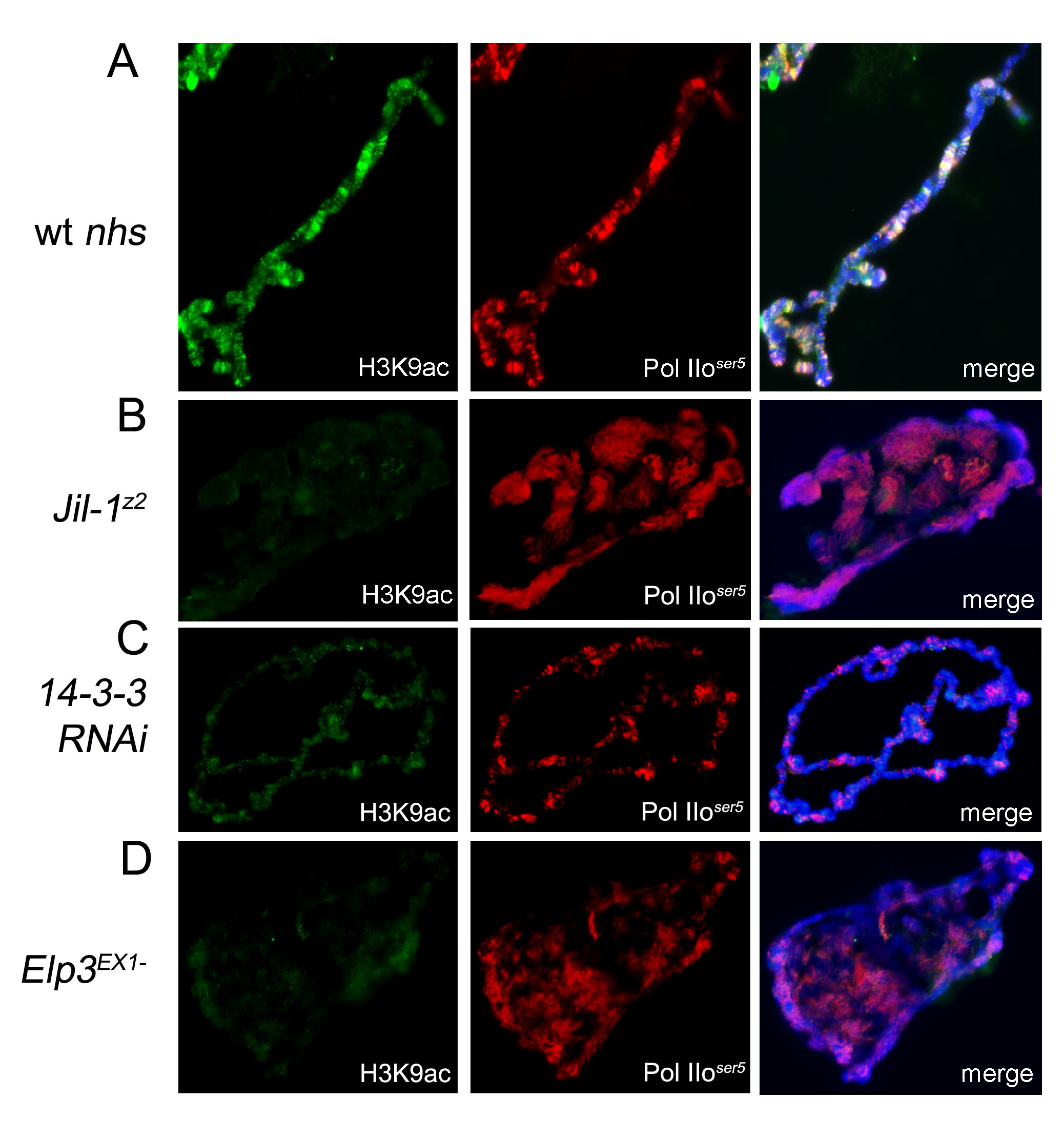


Supporting Figure 5


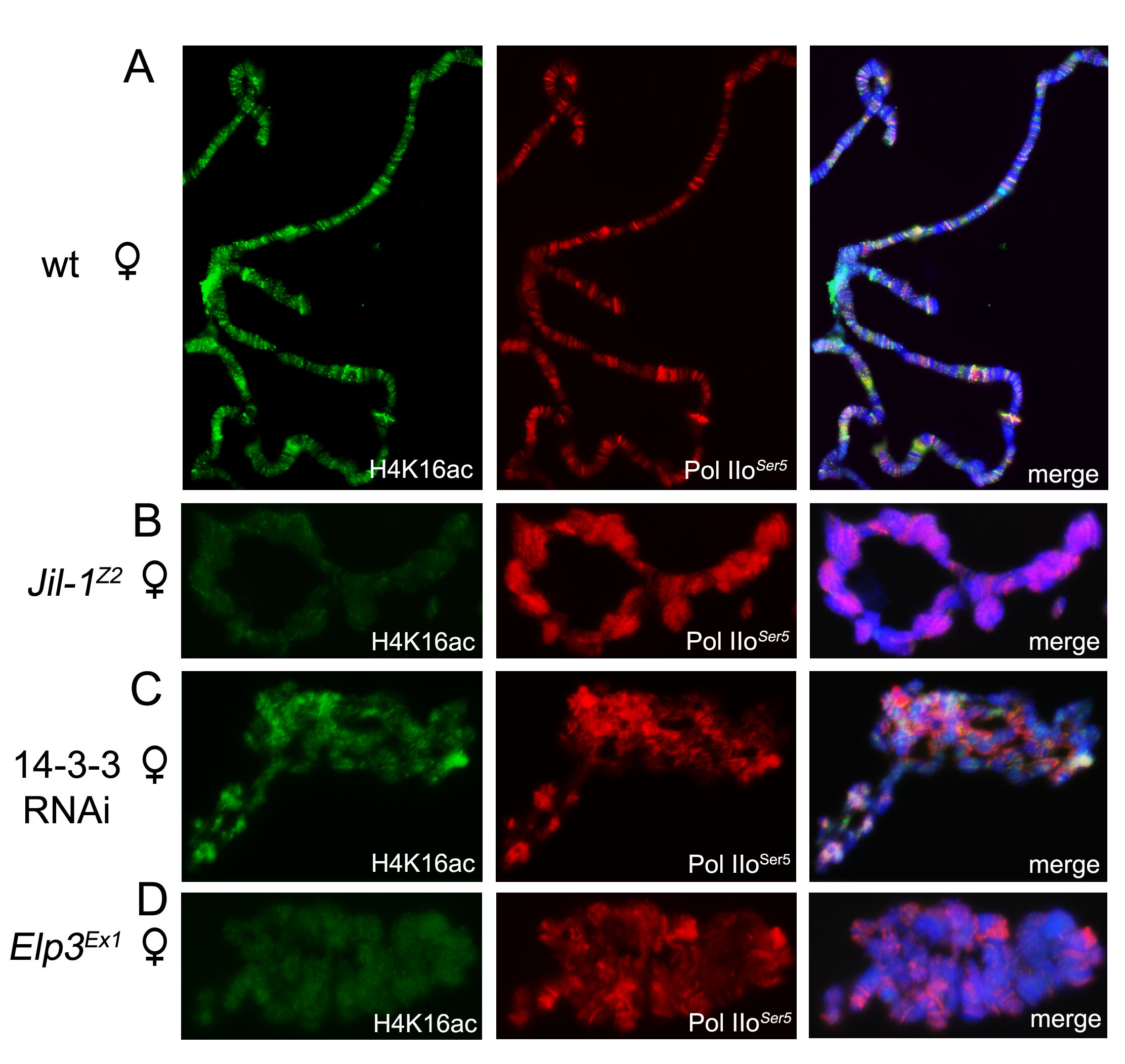


Supporting Figure 6


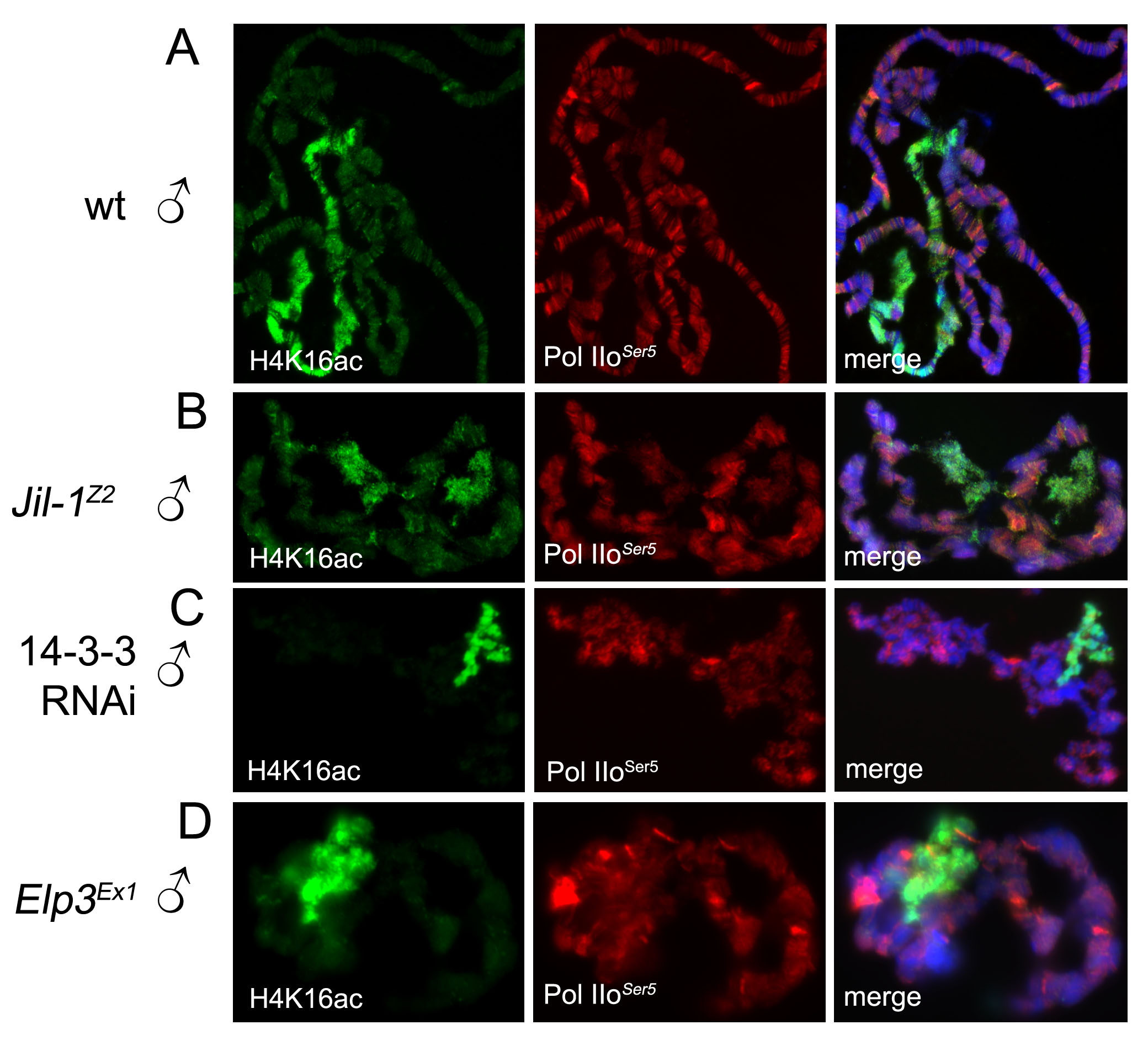


Supporting Figure 7


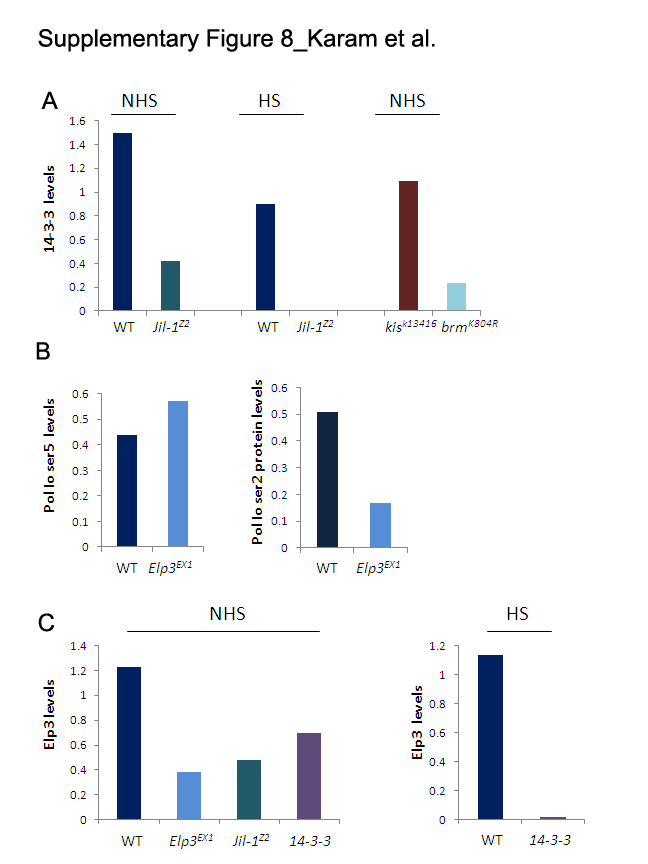


Supporting Figure 8


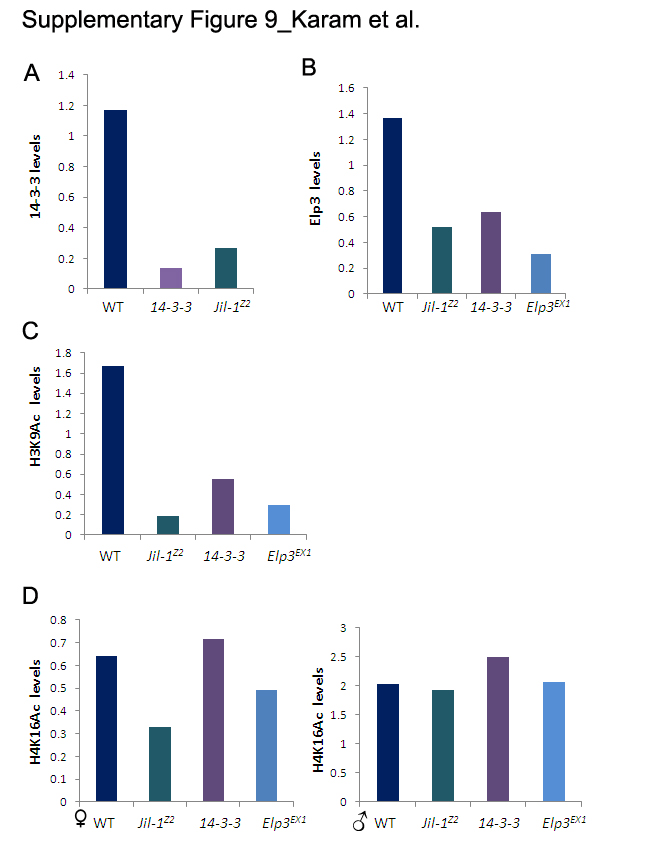


Supporting Figure 9
